# Nutritional, Biophysical and Physiological Characteristics of Wild Rocket Genotypes As Affected by Soilless Cultivation System, Salinity Level of Nutrient Solution and Growing Period

**DOI:** 10.3389/fpls.2017.00300

**Published:** 2017-03-09

**Authors:** Anna Bonasia, Corrado Lazzizera, Antonio Elia, Giulia Conversa

**Affiliations:** Department of the Science of Agriculture, Food and Environment, University of FoggiaFoggia, Italy

**Keywords:** visual quality, antioxidant capacity, nitrate, glucosinulate, vitamin C

## Abstract

With the aim of defining the best management of nutrient solution (NS) in a soilless system for obtaining high quality baby-leaf rocket, the present study focuses on two wild rocket genotypes (“Nature” and “Naturelle”), grown in a greenhouse under two Southern Italy growing conditions—autumn-winter (AW) and winter-spring (WS)—using two soilless cultivation systems (SCS)—at two electrical conductivity values (EC) of NS. The SCSs used were the Floating System (FS) and Ebb and Flow System (EFS) and the EC values were 2.5 and 3.5 dS m^−1^ (EC2.5; EC3.5) for the AW cycle and 3.5 and 4.5 dS m^−1^ (EC3.5; EC4.5) for the WS cycle. The yield, bio-physical, physiological and nutritional characteristics were evaluated. Higher fresh (FY) (2.25 vs. 1.50 kg m^−2^) and dry (DY) (230.6 vs. 106.1 g m^−2^) weight yield, leaf firmness (dry matter, 104.3 vs. 83.2 g kg^−1^ FW; specific leaf area, 34.8 vs. 24.2 g cm^−2^) and antioxidant compounds (vitamin C, 239.0 vs. 152.7 mg kg^−1^ FW; total phenols, 997 vs. 450 mg GAE mg kg^−1^ FW; total glucosinulates-GLSs, 1,078.8 vs. 405.7 mg kg^−1^ DW; total antioxidant capacity-TAC, 11,534 vs. 8,637 μmol eq trolox kg^−1^ FW) and lower nitrates (1,470 vs. 3,460 mg kg^−1^ FW) were obtained under WS conditions. The seasonal differences were evident on the GLS profile: some aliphatic GLSs (gluconapoleiferin, glucobrassicanapin) and indolic 4-OH-glucobrassicin were only expressed in WS conditions, while indolic glucobrassicin was only detected in the AW period. Compared with EFS, FS improved leaf firmness, visual quality, antioxidant content (TAC, +11.6%) and reduced nitrate leaf accumulation (−37%). “Naturelle” performed better than “Nature” in terms of yield, visual quality and nutritional profile, with differences more evident under less favorable climatic conditions and when the cultivars were grown in FS. Compared to EC2.5, the EC3.5 treatment did not affect DY while enhancing firmness, visual quality, and antioxidant compounds (TAC, +8%), and reducing the nitrate content (−47%). The EC4.5 treatment reduced FY and DY and the antioxidant content. Despite seasonal climatic condition variability, FS and the moderate salinity level of NS (3.5 dS m^−1^) can be suggested as optimum.

## Introduction

Wild rocket [*Diplotaxis tenuifolia* (L) DC], also known as arugula or rocket, is a leafy vegetable, belonging to the *Brassicaceae* family, widely consumed in Italy, but with increasing popularity as green salad in other parts of the World. It is characterized by a distinctive flavor, a pungent taste and a wide range of beneficial compounds, contributing to its antioxidant activity (vitamin C, carotenoids, glucosinolates, phenolics) (Hall et al., 2012a; Villatoro-Pulido et al., 2013; Cavaiuolo and Ferrante, 2014). However, it is one of the greatest accumulators of undesirable nitrates among the leafy vegetables and as such is a potential threat to consumer health (Santamaria, 2006).

The quality of leafy vegetables in terms of visual (color, leaf turgidity) and nutritional traits (high phytochemical content, such as antioxidants, and low in anti-nutritional compounds, namely nitrates) depends on several key factors: pedoclimatic factors (such as light, temperature and soil/water salinity), cultural practices (cultivation system, nutrient and water management), and genotype (landraces, cultivar) (Ahuja et al., 2010; Bjorkman et al., 2011).

To fulfill the high year-round demand for this species and to obtain higher qualitative and quantitative yields, standardized culture techniques have been suggested (Sambo et al., 2001). Hence a shift in rocket cultivation from open field to protected cultivation, and in this latter from traditional soil culture to soilless cultivation systems (SCS) is currently occurring in Italy, where an annual cultivation area of about 4,000 hectares under protected cultivation has been reported (Del Grosso, personal communication).

Despite greenhouse conditions, the seasonal climate variability can affect the visual quality and the nutritional profile of the brassica leafy species (Jahangir et al., 2009; Hamilton and Fonseca, 2010; Bjorkman et al., 2011; Soengas et al., 2011). Indeed, warmer and better lighted seasons have been reported to improve total phenol and ascorbic acid contents in *Eruca sativa, Diplotaxis tenuifolia* and *Lepidium sativum* (Hamilton and Fonseca, 2010).

Among the cultivation techniques, SCSs allow better control of plant growth and quality of the product compared with other cultivation methods, through the management of the composition, the temperature, the dissolved oxygen concentration, the electrical conductivity (EC) and the pH of the nutrient solution (NS) (Olympios, 1999).

The floating system (FS) is one of the easiest and cheapest SCS (low installation and manpower costs) used to produce baby-leaf vegetables (Gonnella et al., 2003). As FS is a static hydroponic system, one of the main disadvantages is represented by the limited oxygen level frequently occurring in the NS (Morard and Silvestre, 1996). To cope with this potential limit, the ebb and flow system (EFS) can be used as an alternative for growing baby-leaf vegetables. The two SCSs differ in the distribution of the NS: in FS, roots are constantly submerged in the NS, whereas in EFS, the NS is periodically supplied to the root substrate by sub-irrigation. Only limited information is available on the effect of EFS both on vegetable crop (Rouphael and Colla, 2005) and on rocket salad (Nicola et al., 2003; Hamilton and Fonseca, 2010).

As the EC is indicative of the salinity level of the NS, the EC management of NS results in the management of saline stress on plants. A controlled saline stress can be applied in order to increase the production of secondary metabolites (phytochemicals/antioxidants) and sensorial traits and to reduce anti-nutritional factors, improving the “whole” quality of vegetable product (Francois and Maas, 1994; Gruda, 2009).

Appropriate control of the NS salinity level in SCSs could be successful for rocket leaves. Barbieri et al. (2011) reported an improvement in dry matter content, visual appearance, carotenoids and phenols of *E. sativa*, grown in FS, with the increase of NS salinity up to 50 mM of NaCl (~5 dS m^−1^). According to Hamilton and Fonseca (2010), the increase in NS salinity up to 7.5 dS m^−1^ improves ascorbic acid and phenols in *E. sativa, D. tenuifolia* and *Lepidium sativum*, grown in EFS.

With the aim of defining the best management of NS in SCSs to obtain high quality baby-leaf rocket, the present research was undertaken to investigate the effect on yield, bio-physical, physiological and nutritional proprieties of two wild rocket genotypes grown in FS and EFS at different salinity levels of NS. To account for seasonal variability, trials were performed during the autumn-winter and winter-spring periods.

## Materials and methods

### Crop and treatments

Two experiments were carried out in the autumn-winter (AW) period of 2013 and in the winter-spring (WS) period of 2014 (Supplementary Figure 1), in an unheated greenhouse covered with wavy methyl polymethacrylate (Ondex), located in Foggia (Puglia region, Southern Italy, latitude 41° 46′ N, longitude 15° 55′ E, 74 m a.s.l.).

The experimental factors were (i) two soilless cultivation systems (SCSs): floating system (FS) and ebb and flow system (EFS), (ii) two levels of electrical conductivity (EC) in the nutrient solution (NS): 2.5 dS m^−1^ (EC2.5) and 3.5 dS m^−1^ (EC3.5) in AW trial, and EC3.5 and 4.5 dS m^−1^ (EC4.5) in the WS trial, and (iii) two genotypes of wild rocket: “Naturelle” (Royal Seed) and “Nature” (Coraseed), both belonging to the “Frastagliata” leaf typology.

In both SCSs the set-up consisted of aluminum benches (256 cm long, 96 cm wide, with 5 cm high border). Each bench was connected through a pump to a 100 L water tank positioned below, which was used for NS replenishment or for its movement. Sixteen polystyrene trays each containing 336 cells, were arranged on the bench. The cells, filled with inert substrate (perlite), were sowed with rocket salad multi-seed pellets (each containing ~20 seeds), and were used as containers for plant growth.

In FS, NS was always maintained on the bench (50 L, ~2 cm of water height), excluding a daily movement of NS between the bench and the tank below for oxygen enrichment (emptying and refilling the bench). In EFS, the trays were laid on the benches and were periodically sub-irrigated by a 3 min flow of NS through the benches at the base of trays, five times a day (every 100 min) in the period between 8:00 a.m. and 4:00 p.m. In both cases of a total 50 L of NS was maintained throughout the cycle by replenishment with new NS.

Sowing was carried out on 30 October, 2013 and on 20 January, 2014 obtaining a density of 957 bunches of plants per square meter. Harvest occurred on 12 December, 2013 and 11 March, 2014, 43 and 50 days after sowing in the AW and WS cycles, respectively.

A split-split plot experimental design was adopted with three replications: the soilless cultivation system (SCS) as main plots, the level of EC of NS as sub-plots (one bench with 16 trays), and the genotype as sub-sub-plots (8 trays per genotype on each bench) (the experimental unit).

The concentration of the nutrients was 140, 50, 200, and 100 mg L^−1^ of N, P, K, Ca, Mg, and S, with a NO_3_:NH_4_ ratio of 4:1. A double concentration of microelements was used compared with the Hoagland standard solution. The different salinity levels in the NS were obtained by adding NaCl.

After sowing, trays were kept floating on tap water (pH 6.8 ± 0.2 and EC 0.7 ± 0.2 dS m^−1^) until plant emergence. After emergence, the NS treatments were started using either the FS or EFS management approach. The EC, the dissolved O_2_ and the pH of the NS were checked every 2 days. The pH was maintained between 5.5 and 6.5, through the addition of 1M HCl. The EC and the pH of the NS were measured using a hand-held conductivity- and pH-meter (Hanna Instruments Italia s.r.l., Villafranca, PD, Italy) and the dissolved O_2_ (mg L^−1^) was measured with a hand-held oximeter (Crison Strumenti, Spa, OXI 45+, Carpi-Modena, Italy).

### Data collection and analysis

#### Sampling and measurements

Rocket salad was harvested by cutting the leaf rosette bunches about 1 cm above the collar when plants were at the optimal stage for fresh consumption as baby leaves (less than 10 cm long). The raw material was directly transported to the laboratory (~1 km away) and immediately processed within 1 h after harvest.

Bio-physical and physiological (yield, dry matter content, specific leaf area, main color indices, chlorophyll content, relative water content, electrolytic leakage), and nutritional (vitamin C, ascorbic acid, de-hydro-ascorbic acid, nitrate, carotenoid, glucosinulate and phenol contents, lypophilic, hydrophilic and total antioxidant capacity) parameters of the product were determined. All samples were analyzed in triplicate.

##### Bio-physical and physiological measurements

Fresh weight yield (FY) and dry weight yield (DY) was calculated by considering the whole experimental unit.

After harvest leaves from each plot were well mixed to obtain a homogeneous sample for measurements. Leaf area was measured on a sample of 30 plants for each treatment using LI-COR 3100 (LICOR, Lincoln, NE, USA).

The dry matter concentration (DM) was calculated as dry weight (DW)/fresh weight (FW)^*^100. In order to determine the DW, fresh plant material was dried in a thermo-ventilated oven at 70°C until it reached a constant mass. The Specific Leaf Area (SLA) was expressed as DW/leaf area unit.

The leaf color indices were measured on fresh material using a portable tristimulus color-meter (Minolta Chroma Meter CR-200; Minolta Camera Co. Ltd., Osaka, Japan), using the CIE-L^*^a^*^b^*^ scale 1976. The chroma meter was calibrated using a standard white color, and color was expressed in the tristimulus L^*^ (lightness), a^*^(green to red), and b^*^ (yellow to blue), from which hue angle (h°) was calculated.

The total chlorophyll (CHL) was extracted from previously frozen samples by homogenizing in 80% acetone, spectrophotometrically measured and estimated by the equation of Dere et al. (1998) and expressed on a unit area and on a dry weight basis.

The relative water content (RWC) was determined on fresh leaf blade discs. The sample was first weighed to determine fresh weigh (FW) and then it was hydrated to full turgidity for 24 h, under normal room light and temperature conditions, in de-ionized water in a closed Petri dish. Then the sample was taken out of the water and well dried off with filter paper and immediately weighed to obtain fully turgid weight (TW). The sample was then oven-dried at 70°C and weighed to determine dry weight (DW). The RWC was estimated from the equation reported by Barrs and Weatherley (1962): RWC = (FW– DW)/(TW – DW).

The electrolyte leakage (EL) was determined according to the method of Yan et al. (1996). A portion of fresh leaf material (3 g) was weighed in a glass beaker containing twice-distilled water. The electrical conductivity of the solution (EC1) was measured using a hand-held conductivity-meter (Hanna Instruments Italia s.r.l., Villafranca, PD, Italy). After boiling the sample for 2 min and cooling it to room temperature, the electrical conductivity of the solution was re-measured (EC2). The percentage of electrolyte leakage was calculated as: EL (%) = (EC1/EC2)^*^100.

##### Nutritional measurements

The content of nitrate, chloride, total carotenoids, total phenols, vitamin C and its components and the total antioxidant capacity (TAC) and its components were determined from frozen plant material successively lyophilized and then ground into fine particles.

The nitrate and total phenols contents of were determined as reported in Bonasia et al. (2013). Ascorbic acid (AA) was extracted according to the modified method of Koh et al. (2012). In order to determine the total content of vitamin C (AA+DHAA-dehydroascorbic acid) and indirectly the DHAA content, DHAA was reduced with dithiothreitol (DTT). Reduced samples were also injected into the chromatographic system. The ion chromatography instrument equipment (ICS 3000 System, Dionex) included: a 10 μL injection loop, C18—5 μm reverse-phase ion-exchange columns (Acclaim 120, Dionex) combined with a UV-visible detector (RLSC Diode Array Detector, Dionex). AA was identified and quantified by retention time and spectra. The mobile phase was 0.05 M monobasic potassium phosphate buffer (KH_2_PO_4_) adjusted to pH 4.5 for the first 6 min, gradually followed by buffer and ethanol in a 60:40 ratio from the 6th to the 10th min; 1 min to return to 100% buffer, final 5 min at 100% buffer.

The flow rate was fixed at 1 mL min^−1^; the temperature of the column was set at 30°C. The detection wavelength was 254 nm and the UV spectra were in the 190–350 nm range. The method was calibrated with a curve of standard AA solution.

The TAC was assessed as TEAC (Trolox Equivalent Antioxidant Capacity) according to Re et al. (1999). The hydrophilic fraction (HAC) was extracted twice from samples (30 mg) by 1 mL of 70 % methanol in a shaking water bath (100 rpm, 30°C) for 15 min and by centrifugation (13,000 rpm for 10 min). The supernatants were combined. The lipophilic components (LAC) were extracted twice with 1 mL of hexane, using the above conditions.

Glucosinolate (GLS) extraction and desulphation was carried out were determined as reported in Conversa et al. (2016). In accordance with the ISO protocol (ISO Method 9167-1, 1992), the de-sulphoglucosinolates (d-GLSs) were separated using a gradient HPLC method with an ICS 3000 System (Dionex) using a 10 μL injection loop, C18—5 μm reverse-phase ion-exchange columns (Kinetex Core-Shell, Phenomenex) combined with a UV-visible detector (RLSC Diode Array Detector, Dionex) set to a wavelength of 229 nm. The oven temperature was set at 35°C. Compounds were separated using the following program, with a flow rate of 0.8 mL min^−1^: one minute at 100% H_2_O; 49 min gradient from 0 to 25% (v/v) ethanol; 4 min gradient to return to 100% H_2_O; 10 min at 50% acetonitrile/water (v/v); final 10 min at 100% H_2_O. Individual GLSs were identified by comparing retention times with those of available GLS standards and certified glucosinolate levels of certified reference materials recommended by the E.U. and ISO (BC 367).

Total carotenoids was determined as followed: MgCO_3_ (0.05 g) was added to a 0.1 g of sample to neutralize cytosolic acids; 0.01 g of celite was used for better tissue disruption. The extraction was with 10 ml of ethanol:hexane (4:3 by volume); 1 ml of pyrogallol solution (5%) was added as antioxidant. The mixture was placed in a mechanical shaker for 15 min and then centrifuged at 6,700 rpm for 10 min, the supernatant was collected. The residue was re-extracted; the two extracts were combined and decanted into a 50-mL tube. The supernatant hexane phase was transferred into another tube, and the lower aqueous phase was discarded. To overcome the problem of carotenoid overestimation by the presence of chlorophylls, a saponification step was included during extraction. In brief, an equal volume of 10% methanolic KOH was added to the recovered hexane phase, the mixture was shaken vigorously for 1 min and placed on ice for 15 min. After centrifugation at 6,700 rpm for 10 min, the supernatant (hexane phase) was collected and washed 2 times with 15 ml of NaCl 10% solution and 2 times with 15 ml of water. The aqueous phase was discarded. All samples were stored at −25°C until analysis. The total carotenoids in the extract was measured at 450 nm using a UV-visible spectrophotometer (Shimadzu UV-1800) and estimated according to the “Method of Mean” reported by Biehler et al. (2009).

### Statistical analysis

The statistical processing was carried out using GLM (General Linear Model) procedure—SAS software. The least significant difference (LSD) test (*P* = 0.05) was used to establish differences between means.

## Results and discussion

### Growing season

#### Yield, bio-physical, and physiological characteristics of wild rocket

Wild rocket is a cool-season crop with 2 and 25°C as minimum and maximum temperatures respectively, and with long day-lengths and high temperatures resulting in faster growth rate and development (Hall et al., 2012b).

The climatic data of the two trials, including the internal greenhouse temperature, the solar radiation and day-length, are reported as Supplementary Figure 1. In both experimental trials, mean temperature was quite similar (13.4°C). However, from the mid cycle until the harvest of the autumn-winter (AW), temperatures frequently dropped below 5°C as minimum values. On the contrary, in the winter-spring (WS) cycle, maximum temperatures were frequently higher than 20°C and both solar radiation and day-length increased during the cycle. The cumulated solar radiation was approximately 36% higher in the WS than the AW cycle (429.3 vs. 315.3 MJ m^−2^).

In the WS cycle rocket yield was higher than that in the AW one, both as fresh (FY) (2.25 vs. 1.50 kg m^−2^, on average) and dry (DY) (230.6 vs. 106.1 g m^−2^) yield (Table 1), probably due to the better thermal and light conditions (Supplementary Figure 1) which could have enhanced plant growth.

**Table 1:** **Effect of soilless cultivation system, electrical conductivity of nutrient solution, and genotype on bio-physical and physiological traits in wild rocket**.

**Treatments**	**Fresh weight yield (FY)**	**Dry weight yield (DY)**	**Dry matter concentration (DM)**	**Specific leaf area (SLA)**	L^*^	**h°**	**Chlorophyll**				**Relative water content (RWC)**	**Electrolyte leakage (EL)**
							***a***	***b***	**Total**	**Total**		
	**(kg m^−2^)**	**(g m^−2^)**	**(g kg^−1^ FW)**	**(g DW m^−2^)**			**(**μ**g cm**^**−2**^**)**			**(mg g^−1^ DW)**	**(%)**	**(%)**
**EXP. 1—AUTUMN-WINTER 2013**												
**SCS**^†^												
EFS	1.52 ± 0.08^a^	116.0 ± 4.1^b^	76.5 ± 4.6^b^	22.5 ± 0.9^b^	46.9 ± 0.4^a^	129.6 ± 0.2^a^	16.5 ± 0.8^b^	3.9 ± 0.2^b^	20.6 ± 1.0^b^	8.7 ± 0.6^b^	79.6 ± 0.8^a^	11.6 ± 2.1^a^
FS	1.38 ± 0.09^a^	124.1 ± 3.7^a^	89.9 ± 4.5^a^	25.9 ± 1.0^a^	45.9 ± 0.3^b^	129.6 ± 0.3^a^	20.5 ± 0.7^a^	5.3 ± 0.3^a^	25.9 ± 1.0^a^	9.6 ± 0.7^a^	79.6 ± 0.6^a^	12.1 ± 1.8^a^
**Salinity level (EC)**												
2.5 dS·m^−1^	1.66 ± 0.09^a^	124.8 ± 3.0^a^	75.2 ± 5.1^b^	21.9 ± 0.7^b^	47.2 ± 0.4^a^	129.0 ± 0.3^b^	16.7 ± 0.9^b^	4.1 ± 0.3^b^	21.0 ± 1.2^b^	9.3 ± 0.8^a^	80.8 ± 0.8^a^	13.6 ± 1.6^a^
3.5 dS·m^−1^	1.33 ± 0.08^b^	121.3 ± 5.0^a^	91.2 ± 4.1^a^	26.5 ± 1.4^a^	45.6 ± 0.3^b^	130.0 ± 0.2^a^	20.5 ± 0.5^a^	5.2 ± 0.2^a^	25.9 ± 0.7^a^	9.0 ± 0.4^a^	78.4 ± 0.6^b^	10.1 ± 2.1^a^
**Genotypes (G)**												
Nature	1.37 ± 0.07^b^	116.6 ± 4.2^b^	85.1 ± 4.5^a^	25.0 ± 0.9^a^	46.9 ± 0.3^a^	129.3 ± 0.2^b^	18.6 ± 0.8^a^	4.7 ± 0.3^a^	23.4 ± 1.1^a^	8.6 ± 0.7^a^	78.9 ± 0.6^b^	10.1 ± 1.8^a^
Naturelle	1.64 ± 0.10^a^	133.3 ± 3.8^a^	81.3 ± 4.9^a^	23.4 ± 1.1^a^	45.9 ± 0.3^b^	130.0 ± 0.3^a^	18.5 ± 0.8^a^	4.6 ± 0.3^a^	23.2 ± 1.1^a^	9.7 ± 0.5^a^	80.3 ± 0.8^a^	13.6 ± 1.8^a^
**Significance**^††^												
SCS	NS	^*^	^*^	^*^	^*^	NS	^***^	^***^	^***^	^*^	NS	NS
EC	^*^	NS	^*^	^*^	^**^	^***^	^***^	^**^	^***^	NS	^*^	NS
G	^*^	^*^	NS	NS	^*^	^*^	NS	NS	NS	NS	^*^	NS
SCSxEC	NS	NS	NS	NS	NS	NS	NS	NS	NS	NS	NS	NS
GxSCS	NS	NS	NS	NS	NS	NS	NS	NS	NS	NS	NS	NS
GxEC	NS	NS	NS	NS	NS	NS	NS	NS	NS	NS	NS	NS
GxECxSCS	NS	NS	NS	NS	NS	NS	NS	NS	NS	NS	NS	NS
**EXP. 2—WINTER-SPRING 2014**												
**SCS**^†^												
EFS	2.22 ± 0.10^a^	210.7 ± 6.2^b^	94.9 ± 3.5^b^	32.9 ± 0.6^b^	55.7 ± 0.2^a^	114.7 ± 0.1^b^	10.0 ± 0.1^b^	4.6 ± 1.0^b^	14.9 ± 1.1^b^	5.0 ± 0.2^b^	66.3 ± 4.4^a^	16.1 ± 2.0^a^
FS	2.20 ± 0.11^a^	250.4 ± 9.8^a^	113.8 ± 3.8^a^	36.7 ± 0.7^a^	54.0 ± 0.2^b^	115.9 ± 0.2^a^	13.0 ± 0.1^a^	6.3 ± 0.5^a^	19.6 ± 0.6^a^	5.8 ± 0.4^a^	62.8 ± 5.2^a^	20.2 ± 2.6^a^
**Salinity level (EC)**												
3.5 dS·m^−1^	2.33 ± 0.09^a^	239.3 ± 4.1^a^	102.7 ± 4.1^a^	34.3 ± 1.0^a^	54.9 ± 0.2^a^	115.2 ± 0.2^a^	12.0 ± 0.1^a^	5.9 ± 0.3^a^	18.3 ± 0.4^a^	5.6 ± 0.3^a^	63.9 ± 5.5^a^	19.4 ± 2.6^a^
4.5 dS·m^−1^	2.09 ± 0.10^b^	221.5 ± 12.2^b^	106.0 ± 3.3^a^	35.9 ± 0.2^a^	54.9 ± 0.2^a^	115.4 ± 0.2^a^	11.0 ± 0.1^b^	4.9 ± 0.5^b^	16.2 ± 0.6^b^	4.9 ± 0.3^b^	64.9 ± 4.4^a^	16.8 ± 2.2^a^
**Genotypes (G)**												
Nature	1.86 ± 0.10^b^	195.3 ± 9.6^b^	105.0 ± 3.3^a^	35.2 ± 0.7^a^	54.8 ± 0.2^a^	115.2 ± 0.2^a^	11.3 ± 0.1^b^	5.0 ± 0.6^b^	16.6 ± 0.7^b^	5.0 ± 0.2^b^	65.5 ± 5.3^a^	16.4 ± 2.2^a^
Naturelle	2.57 ± 0.09^a^	266.5 ± 7.5^a^	103.7 ± 4.2^a^	34.5 ± 0.7^a^	54.9 ± 0.3^a^	115.4 ± 0.2^a^	11.6 ± 0.1^a^	5.8 ± 0.1^a^	17.7 ± 0.2^a^	5.9 ± 0.4^a^	63.5 ± 4.7^a^	19.5 ± 2.5^a^
**Significance**^††^												
SCS	NS	^*^	^***^	^*^	^***^	^***^	^***^	^*^	^**^	^*^	NS	NS
EC	^*^	^*^	NS	NS	NS	NS	^*^	^*^	^*^	^*^	NS	NS
G	^**^	^*^	NS	NS	NS	NS	^*^	^*^	^*^	^*^	NS	NS
SCSxEC	NS	NS	NS	NS	NS	NS	^*^	^*^	^*^	NS	NS	NS
GxSCS	NS	NS	NS	NS	^**^	NS	^*^	^*^	^*^	NS	NS	NS
GxEC	NS	NS	NS	NS	NS	NS	NS	NS	NS	NS	NS	NS
GxECxSCS	NS	NS	NS	NS	NS	NS	NS	NS	NS	NS	NS	NS

† *SCS is Soilless Cultivation System: EFS, Ebb and Flow system; FS, Floating System*.

†† *NS*,

*, **, and ***, *not significant or significant at P ≤ 0.05, P ≤ 0.01, or P ≤ 0.001, respectively*.

a, b *Means (±SE of mean) in columns not sharing the same letters are significantly different according to LSD test (P = 0.05)*.

Under WS compared with AW conditions, higher dry matter concentration (DM) (104.3 vs. 83.2 g kg^−1^ FW, on average) and specific leaf area (SLA) (34.8 vs. 24.2 g cm^−2^, on average) were obtained, both parameters indicating more thickened leaves. Similar behavior for DM and SLA has also been reported in butterhead lettuce leaves grown during different seasons (Bonasia et al., 2013).

In the WS trial, the leaves had a paler color as indicated by the higher values of the brightness index (L^*^) (54.9 vs. 46.4, on average) and the lower values of hue angle (h°) (115.3° vs. 129.6°, on average) (Table 1). Clearly the differences in leaf color were consistent with the differences in CHL content, being lower in the WS than in the AW cycle (17.2 vs. 23.3 μg cm^−2^ or 5.4 vs. 9.2 mg g^−1^ on DW basis, on average) (Table 1). Similarly, paler leaves have been reported in spinach when grown under high temperature and sunlight availability (March), compared with those obtained in a colder period with less light (January) (Conte et al., 2008).

The WS rocket leaves compared to the AW product also had more damaged membranes and more dehydrated tissues as pointed out by the greater membrane efflux of electrolytes (EL) (17.9 vs. 11.8%, on average) and by the lower relative water content (RWC) (64.5 vs. 79.6%, on average) (Table 1). This physiological status of the leaves could be linked to the less favorable climatic conditions of the WS trial (Supplementary Figure 1).

#### Nutritional traits of wild rocket

Wild rocket product, obtained during WS period, with increased temperature and sunlight (Supplementary Figure 1), had an improved nutritional profile linked to a higher content of antioxidant compounds (Tables 2, 3), only with the exception of the concentration of carotenoids, being lower in the WS than in AW grown leaves (7.7 vs. 12.8 mg 100 g^−1^ FW) (Table 2).

**Table 2:** **Effect of soilless cultivation system, salinity level, and genotype on nutritional traits of wild rocket**.

**Treatments**	**Chloride**	**Nitrate**	**Total carotenoids**	**Total phenols**	**Ascorbic acid**	**De-hydro-ascorbic acid**	**Vitamin C**	**Antioxidant capacity**		
								**lipophilic**	**hydrophilic**	**total**
	**(mg kg^−1^ DW)**	**(mg kg^−1^ FW)**	**(mg kg^−1^ FW)**	**(mg GAE kg^−1^ FW)^‡^**	**(mg kg**^**−1**^ **FW)**			**(μmol eq trolox kg**^**−1**^ **FW)**		
**EXP. 1—AUTUMN-WINTER 2013**										
**SCS**^†^										
EFS	15,421 ± 1190^a^	4,027 ± 220^a^	113.8 ± 3.2^b^	433 ± 11.3^b^	76.7 ± 1.1^b^	59.6 ± 2.0^b^	136.3 ± 3.1^b^	165 ± 16^a^	8,138 ± 400^b^	8,323 ± 416^b^
FS	11,796 ± 856^b^	2,895 ± 198^b^	142.9 ± 8.6^a^	468 ± 8.7^a^	80.0 ± 1.2^a^	89.2 ± 15.0^a^	169.2 ± 16.2^a^	185 ± 30^a^	8,786 ± 125^a^	8,951 ± 155^a^
**Salinity level (EC)**										
2.5 dS·m^−1^	12,176 ± 803^b^	4,524 ± 200^a^	120.2 ± 6.9^b^	431 ± 11.3^b^	64.9 ± 1.2^b^	65.8 ± 3.0^b^	130.8 ± 4.2^b^	209 ± 27^a^	8,083 ± 425^b^	8,293 ± 452^b^
3.5 dS·m^−1^	15,042 ± 1365^a^	2,397 ± 215^b^	138.0 ± 4.9^a^	470 ± 8.0^a^	91.8 ± 1.0^a^	83.0 ± 14.0^a^	174.7 ± 15.0^a^	141 ± 17^a^	8,841 ± 100^a^	8,982 ± 117^a^
**Genotypes (G)**										
Nature	14,428 ± 1434^a^	3,237 ± 215	120.7 ± 4.2^b^	446 ± 9.6^a^	79.0 ± 1.1^a^	83.5 ± 7.0^a^	162.5 ± 8.1^a^	167 ± 25^a^	7,767 ± 215^b^	7,934 ± 240^b^
Naturelle	12,789 ± 926^a^	3,684 ± 200^a^	137.5 ± 9.6^a^	454 ± 12.2^a^	77.7 ± 1.3^a^	65.4 ± 12.0^a^	143.0 ± 13.3^a^	183 ± 24^a^	9,157 ± 324^a^	9,340 ± 348^a^
**Significance**^††^										
SCS	^*^	^*^	^***^	^*^	^*^	^*^	^*^	NS	^*^	^*^
EC	^*^	^**^	^***^	^**^	^*^	^*^	^*^	NS	^*^	^*^
G	NS	^*^	^*^	NS	NS	NS	NS	NS	^*^	^*^
SCSxEC	NS	NS	NS	NS	NS	NS	NS	NS	NS	NS
GxSCS	NS	^*^	NS	NS	NS	NS	NS	NS	NS	NS
GxEC	NS	NS	NS	NS	NS	NS	NS	NS	NS	NS
GxECxSCS	NS	NS	NS	NS	NS	NS	NS	NS	NS	NS
**EXP. 2—WINTER-SPRING 2014**										
**SCS**^†^										
EFS	39,849 ± 4,679^a^	2,026 ± 120^a^	66.0 ± 4.1^b^	990 ± 75^a^	23.8 ± 1.2^b^	175.5 ± 28.0^b^	199.8 ± 29.2^b^	625 ± 18^a^	10,112 ± 692^b^	10,737 ± 710^b^
FS	24,330 ± 3,850^b^	919 ± 128^b^	89.1 ± 3.9^a^	1,010 ± 75^a^	35.2 ± 1.2^a^	243.5 ± 5.0^a^	279.3 ± 6.2^a^	564 ± 45^a^	11,767 ± 700^a^	12,330 ± 745^a^
**Salinity level (EC)**										
3.5 dS·m^−1^	27,439 ± 3,198^b^	1,491 ± 125^a^	78.6 ± 3.5^a^	929 ± 65^a^	28.1 ± 1.5^a^	218.3 ± 2.0^a^	246.5 ± 3.5^a^	578 ± 25^a^	10,844 ± 638^a^	11,422 ± 663^a^
4.5 dS·m^−1^	36,739 ± 5,234^a^	1,454 ± 130^a^	76.5 ± 3.9^a^	1,072 ± 85^a^	31.7 ± 1.5^a^	188.9 ± 18.0^b^	222.0 ± 19.5^b^	610 ± 35^a^	11,035 ± 900^a^	11,645 ± 935^a^
**Genotypes (G)**										
Nature	31,605 ± 4,237^a^	1,211 ± 125^b^	76.2 ± 4.3^a^	1,026 ± 72^a^	33.3 ± 1.3^a^	225.5 ± 35.0^a^	259.3 ± 36.3^a^	602 ± 28^a^	11,212 ± 859^a^	11,815 ± 887^a^
Naturelle	32,573 ± 4,500^a^	1,734 ± 131^a^	78.9 ± 3.3^a^	974 ± 75^a^	25.6 ± 1.3^a^	192.5 ± 5.0^a^	218.7 ± 6.3^a^	586 ± 35^a^	10,667 ± 688^a^	11,253 ± 723^a^
**Significance**^††^										
SCS	^***^	^**^	^***^	NS	^*^	^**^	^***^	NS	^*^	^*^
EC	^**^	NS	NS	NS	NS	^*^	^*^	NS	NS	NS
G	NS	^*^	NS	NS	NS	NS	NS	NS	NS	NS
SCSxEC	NS	NS	NS	NS	NS	NS	NS	NS	NS	NS
GxSCS	NS	^*^	NS	NS	NS	NS	NS	NS	NS	NS
GxEC	NS	NS	NS	NS	NS	NS	NS	NS	NS	NS
GxECxSCS	NS	NS	NS	NS	NS	NS	NS	NS	NS	NS

† *SCS is Soilless Cultivation System: EFS, Ebb and Flow system; FS, Floating System*.

†† *NS*,

*, **, and ***, *not significant or significant at P ≤ 0.05, P ≤ 0.01, or P ≤ 0.001, respectively*.

a, b *Means (±SE of mean) in columns not sharing the same letters are significantly different according to LSD test (P = 0.05)*.

‡ *GAE, gallic acid equivalent*.

**Table 3:** **Effect of soilless cultivation system, salinity level, and genotype on glucosinulate profile in wild rocket**.

**Treatments**	**Total GLS**	**Progoidrin (PRO)**	**Glucorafanin (GRAF)**	**Epiprogoitrin (EPRO)**	**Glucoerucin (GLUE)**	**Glucobrassicin (GBC)**	**Gluconapoleiferin (GNL)**	**Glucobrassicanapin (GBN)**	**4-hydroxy-glucobrassicin (4-OH)**
**(mg kg**^**−1**^ **DW)**									
**EXP. 1—AUTUMN-WINTER 2013**									
**SCS**^†^									
EFS	299.3 ± 60.7^b^	43.7 ± 28.1^a^	235.4 ± 25.1^a^	6.8 ± 2.1^b^	12.7 ± 2.2^b^	0.61 ± 0.2^b^	n.d.	n.d.	n.d.
FS	512.2 ± 100.1^a^	14.1 ± 7.2^a^	397.0 ± 75.1^a^	30.2 ± 3.2^a^	70.1 ± 4.1^a^	30.4 ± 10.5^a^	n.d.	n.d.	n.d.
**Salinity level (EC)**									
2.5 dS·m^−1^	434.2 ± 82.1^a^	42.1 ± 30.2^a^	338.6 ± 45.3^a^	14.5 ± 3.2^b^	38.1 ± 3.3^b^	0.49 ± 0.10^b^	n.d.	n.d.	n.d.
3.5 dS·m^−1^	378.3 ± 92.7^a^	15.3 ± 4.1^a^	293.8 ± 70.0^a^	22.5 ± 4.5^a^	44.8 ± 3.1^a^	30.5 ± 11.0^a^	n.d.	n.d.	n.d.
**Genotypes (G)**									
Nature	320.0 ± 70.3^b^	15.4 ± 2.0^a^	273.7 ± 50.1^b^	14.9 ± 5.1^a^	15.4 ± 2.1^b^	30.3 ± 11.0^a^	n.d.	n.d.	n.d.
Naturelle	491.5 ± 73.6^a^	42.4 ± 33.0^a^	358.7 ± 33.2^a^	22.1 ± 3.1^a^	67.5 ± 4.1^a^	0.76 ± 0.2^b^	n.d.	n.d.	n.d.
**Significance**^††^									
SCS	^*^	NS	NS	^*^	^*^	^*^	−	−	−
EC	NS	NS	NS	^*^	^*^	^*^	−	−	−
G	^*^	NS	^*^	NS	^*^	^*^	−	−	−
SCSxEC	NS	NS	NS	NS	NS	NS	−	−	−
GxSCS	NS	NS	^*^	^*^	^*^	NS	−	−	−
GxEC	NS	NS	NS	NS	NS	NS	−	−	−
GxECxSCS	NS	NS	NS	NS	NS	NS	−	−	−
**EXP. 2—WINTER-SPRING 2014**									
**SCS**^†^									
EFS	1,012 ± 57.8^b^	175.8 ± 5.0^b^	396.0 ± 32.4^a^	42.0 ± 2.1^a^	229.8 ± 5.2^b^	n.d.	111.4 ± 8.1^a^	44.6 ± 3.5^b^	12.3 ± 1.5^b^
FS	1,122 ± 46.5^a^	224.1 ± 1.2^a^	389.2 ± 25.4^a^	9.7 ± 1.0^b^	266.7 ± 4.5^a^	n.d.	98.7 ± 10.2^a^	60.7 ± 2.0^a^	72.7 ± 2.5^a^
**Salinity level (EC)**									
3.5 dS·m^−1^	1,170 ± 61.2^a^	161.5 ± 5.3^b^	538.9 ± 29.0^a^	39.5 ± 2.2^a^	246.1 ± 5.1^a^	n.d.	131.2 ± 11.1^a^	22.3 ± 4.0^b^	30.5 ± 4.5^b^
4.5 dS·m^−1^	978 ± 43.9^b^	337.1 ± 1.3^a^	259.2 ± 28.1^b^	12.1 ± 2.1^b^	251.9 ± 5.1^a^	n.d.	80.7 ± 6.3^b^	80.9 ± 0.5^a^	56.2 ± 0.5^a^
**Genotypes (G)**									
Nature	883 ± 37.1^b^	100.9 ± 1.2^b^	364.1 ± 20.8^b^	38.2 ± 2.1^a^	180.7 ± 5.1^b^	n.d.	83.4 ± 3.4^b^	101.2 ± 3.5^a^	14.3 ± 1.0^b^
Naturelle	1,275 ± 61.7^a^	311.3 ± 4.9^a^	423.0 ± 38.4^a^	10.7 ± 1.1^b^	324.4 ± 5.2^a^	n.d.	128.3 ± 10.1^a^	2.5 ± 0.5^b^	76.5 ± 3.5^a^
**Significance**^††^									
SCS	^*^	^*^	NS	^*^	^*^	−	NS	^*^	^*^
EC	^*^	^*^	^*^	^*^	NS	−	^*^	^*^	^*^
G	^*^	^*^	^*^	^*^	^*^	−	^*^	^*^	^*^
SCSxEC	NS	NS	NS	NS	NS	−	NS	NS	NS
GxSCS	NS	NS	NS	NS	NS	−	NS	NS	NS
GxEC	NS	NS	NS	NS	NS	−	NS	NS	NS
GxECxSCS	NS	NS	NS	NS	NS	−	NS	NS	NS

† *SCS is Soilless Cultivation System: EFS, Ebb and Flow system; FS, Floating System*.

†† NS and

* *not significant or significant at P ≤ 0.05, respectively*.

a, b *Means (±SE of mean) in columns not sharing the same letters are significantly different according to LSD test (P = 0.05)*.

With regard to antioxidant compounds, under the WS, compared with the AW conditions, rocket leaves had higher vitamin C (239.0 vs. 152.7 mg kg^−1^ FW, on average), and its main component de-hydro-ascorbic acid (DHAA), total phenol (TP) (997 vs. 450 mg GAE mg kg^−1^ FW, on average) (Table 2), and total glucosinolate (GLS) concentration (1,078.8 vs. 405.7 mg kg^−1^ DW, on average) (Table 3). Consequently, a higher total antioxidant capacity (TAC) (11,534 vs. 8,637 μmol eq trolox kg^−1^ FW, on average) (Table 2), which measures the efficiency of all antioxidant compounds in scavenging free radicals, was observed in the WS compared with the AW grown leaves. The increase in TAC is mainly due to the enhancement of antioxidant capacity of the hydrophilic components (HAC) (10,939 vs. 8,462 μmol eq trolox kg^−1^ FW, on average) (Table 2), strongly linked to the TP, GLS, and vitamin C contents.

The higher temperature and light conditions in the WS cycle (Supplementary Figure 1), by triggering the secondary metabolism as a plant response, could have elicited the production of these antioxidant compounds.

Several authors have observed higher concentrations of phenolic compounds in broccoli grown under better light conditions (Vallejo et al., 2003; Jahangir et al., 2009), as well as a higher total GLS concentration in *Brassicaceae* vegetables grown under higher light intensity, temperatures and day-length (spring season) have been reported (Jahangir et al., 2009; Bjorkman et al., 2011). In our research the GLS profile of rocket salad leaves showed changes in GLS quality between the two growing seasons (Table 3), as also observed in several studies on rocket salad (Velasco et al., 2007; Hamilton and Fonseca, 2010). In both trials, aliphatic proidrin (PRO), epiproidrin (EPRO), glucoerucin (GLUE) and glucoraphain (GRAF) were detected. However, PRO and GLUE were particularly lower in the AW cycle, when indolic glucobrassicin (GBC) was also detected. Aliphatic glucobrassiconapoleiferin (GNL), glucobrassiconapin (GBN), and indolic 4-hydroxy glucobrassicin (4-OH) were only detected in the WS cycle (Table 3).

The most abundant GLSs were GRAF in both cycles as well as GLUE and PRO in the WS cycle. Similarly, high contents of GRAF (Pasini et al., 2011) and GLUE (Barillari et al., 2005) have also been reported in *E. sativa*.

Vitamin C is represented by the DHAA and the ascorbid acid (AA) fractions. The latter is the main biologically active form of vitamin C, which is essential in the defense against environmentally induced oxidative stress.

AA interacting with the damaging free radicals is subjected to oxidation to de-hydro-ascorbic acid (DHAA). Therefore, the amount of DHAA in the cells is associated with the responsiveness to factors such as light, temperature, salinity and drought (Davey et al., 2000; Locato et al., 2013).

In this study the higher DHAA level in the WS product, representing the most abundant part of the total Vitamin C (Table 2), could be considered further evidence of the plant physiological/metabolic response to the climatic conditions (light and temperature) during the WS cycle (Supplementary Figure 1).

The lipophilic antioxidant capacity (LAC), represented by antioxidant compounds contributing to TAC such as carotenoids and vitamin E, were higher in WS than AW-plants. As carotenoids were lower in the WS grown leaves (Table 2), other compounds could be associated with the increase in LAC.

The mean value of TAC (10,085 μmol eq trolox kg^−1^ FW) was greater than that reported for other species classified as having a high TAC content (>9,000 μmol eq trolox kg^−1^ FW), such as blueberry, cranberry and artichoke (Pennington and Fisher, 2009), so indicating wild rocket as a highly valuable and healthy food.

Moreover, the climatic conditions (Supplementary Figure 1) reduced the anti-nutritional profile of wild rocket product. Indeed, the higher light intensity conditions in the WS compared to the AW period resulted in a lower accumulation of nitrates in the leaves (1,470 vs. 3,460 mg kg^−1^ FW, on average) (Table 2). Similar results have been reported in rocket (Podetta et al., 2011), in lettuce (Bonasia et al., 2013), and in spinach leaf (Conte et al., 2008). It is well-known that light intensity is positively correlated to higher nitrate reductase activity (Blom-Zandstra, 1989). In any case the observed leaf nitrate accumulation (2,466 mg kg^−1^ FW, on average) (Table 2) is much lower than the nitrate limit imposed for rocket salad by European Community Regulation 1258/2011 (7,000 mg kg^−1^ FW).

### Soilless cultivation system (SCS)

#### Yield, bio-physical and physiological traits of wild rocket

In both cycles, while rocket fresh weight yield (FY) was not affected by SCS (Table 1). However, The dry weight yield (DY) was higher in FS than in EFS (187.2 vs. 163.5 g m^−2^ in the WS cycle, and 2.5 vs. 2.1 g m^−2^ in the AW cycle) (Table 1). Consequently in FS a higher dry matter concentration (DM) (+17.5 and + 19.9% in the AW and WS cycles, respectively) and a higher specific leaf area (SLA) (+15.1 and +11.5% in the AW and WS cycles, respectively) were detected (Table 1), resulting in more thickened leaves. In both cycles, FS enhanced the content of chlorophyll pigments expressed both on a leaf area basis (+26 and +31% in the AW and WS cycles, respectively) and on a DW basis (+10 and +16% in the AW and WS cycles, respectively) (Table 1). Consistently, the instrumental color measurements confirmed a generally greener color of leaves in FS, as can be seen from the lower L^*^ (−2% and −3% in the AW and WS cycles, respectively), and higher h° (+1% in the WS cycle) (Table 1). All these aspects contributed to the better firmness and visual quality of the FS compared with EFS product.

In both cycles the lower DY and chlorophyll accumulation (Table 1) in EFS was concomitant with a higher chloride accumulation in EFS compared with FS grown leaves (27,650 vs. 18,000 mg kg^−1^ DW on average) (Table 2).

The inhibition exerted by the chloride ions on the activity of the enzymes involved in the N metabolism, such as NR, NiR, GS, and GDH, is well-known (Barber et al., 1989; Debouba et al., 2006, 2007). Therefore, it can be hypothesized that the high concentration of chloride occurring in the vacuoles of the EFS leaves (Table 2) could have reduced N assimilation and, consequently, plant growth (Table 1). Debouba et al. (2007) also reported a decrease in growth and in dry matter in tomato seedlings as a result of NiR activity inhibition with a concomitant increase in ${\text{NH}}_{4}^{+}$ ions in leaves and roots.

It is likely that the higher leaf chloride accumulation in EFS compared with FS (Table 2) was favored by the NS management. In FS the roots were in the NS throughout the entire crop cycle and the composition of the NS was far more stable over this period. On the contrary, in EFS the NS management (3-min long wettings every 100 min, in the period between 8:00 a.m. and 4:00 p.m.) caused a temporary, but frequent rise in salt concentration of NS in the root zone as a consequence of the partial drying of the limited substrate volume (7 mL) between the intermittent wettings.

No difference emerged between the two SCSs regarding the physiological status of rocket tissue measured through RWC (72.5%, on average) and EL (14.9%, on average) (Table 1), pointing out that the two SCSs were similar in terms of plant water availability and in inducing oxidative stresses on cell membranes.

#### Nutritional traits of wild rocket

The product grown in EFS compared with FS showed a lower concentration of total carotenoids, vitamin C (both in the AA and DHAA components) (Table 2), total GLSs (Table 3), total phenols (only in the AW cycle), and a lower TAC and HAC (Table 2). The decrease in total GLS content was consistent with the decrease of the aliphatic GLUE (in both cycles), with the indolic EPRO and GBC in the AW-leaves, and with the aliphatic GBN and PRO, and the indolic 4-OH in the WS-leaves (Table 3).

The reasons for the differences in antioxidant compound levels between FS and EFS could be due to the higher chloride concentration in the EFS-leaves (Table 2), the antioxidant properties of leafy vegetables being reported to be relatively sensitive to chloride (Xu et al., 1999).

These results are also in agreement with the notable reduction in vitamin C content following a chloride application to the root medium reported in Chinese cabbage (Yin et al., 1989) and in lettuce (Wei et al., 1989).

Moreover, FS allowed a lower leaf nitrate accumulation compared with EFS (−28% and −54.6% in the AW and WS cycles, respectively) (Table 2). The higher chloride leaf accumulation in EFS compared with FS leaves (Table 2) probably could have negatively affected nitrogen assimilation, as above supposed, resulting in a higher accumulation of nitrates in the tissues.

### Electrical conductivity (EC) of nutrient solution (NS)

#### Yield, bio-physical, and physiological traits of wild rocket

In the AW cycle, the EC3.5 compared with the EC2.5 grown leaves showed a lower FY (−18.7%), with no effect on DY (123.0 g m^−2^, on average) (Table 1). Moreover, EC3.5 enhanced DM concentration (+21.3%) and SLA (+21.0%) compared with EC2.5 (Table 1), thus resulting in more thickened leaves.

Passing from EC2.5 to EC3.5 it seems that an osmotic stress occurred. It is well known, indeed, that an increase in the EC in the cultivation medium, within low/moderate threshold values, increases leaf DM concentration (Barbieri et al., 2011), while decreasing crop growth/yield (Maas and Hoffman, 1977; Maas, 1986) due to the osmotic effect caused by the low water potential of the NS (Munns and Termaat, 1986; Jacoby, 1994). In addition, the RWC value of EC3.5-leaves also underlines less hydrated tissues than those grown with EC2.5 (Table 1), thus confirming the negative correlation between RWC and the salinity level of the NS reported by Garrido et al. (2014) and Taârit et al. (2012).

No difference in EL values (11.8%) was observed between the EC levels (Table 1), indicating that the EC3.5 did not affect the integrity of cell membranes as much as the lower EC level (EC2.5).

EC3.5 compared with EC2.5 resulted in higher content of CHL pigments, on an area basis only (+23%) (Table 1), and in more intense green color of leaves, related to the higher h° and the lower L^*^ values (Table 1), supporting a better visual quality of the EC3.5 grown product.

In the WS cycle, the EC3.4- was less productive than EC3.5-crop in terms both of FY (−10.3%) and DY (−7.4%) (Table 1). Moreover, EC4.5 grown leaves did not show any improvement in leaf DM concentration (104.3 g kg^−1^ FW, on average) or thickness (SLA, 35.1 g m^−2^, on average) compared with the EC3.5 grown ones (Table 1).

**Figure 1:**
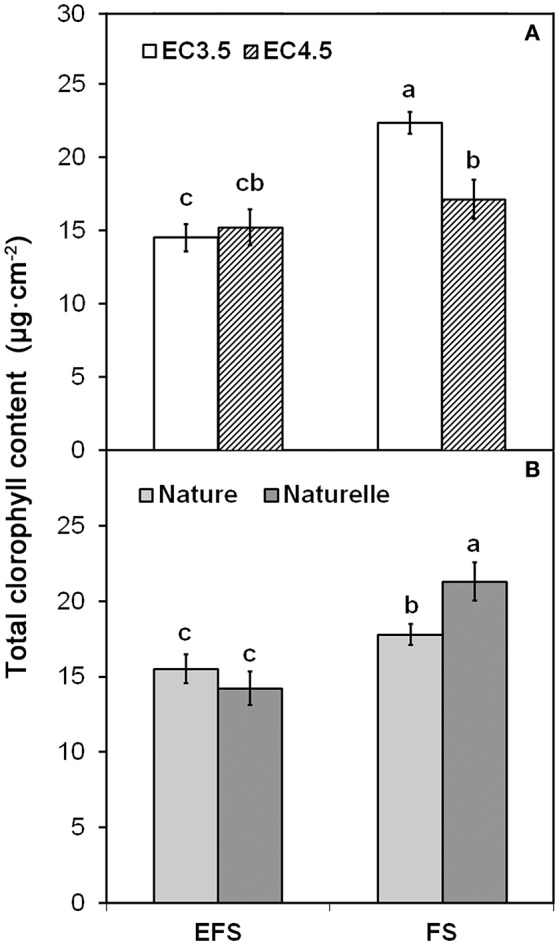
**Effect of soilless cultivation system (FL, Floating system; EFS, Ebb and Flow System) and of salinity level of nutrient solution (EC) (A)** and of genotype **(B)**, on total chlorophyll content of wild rocket leaves, grown in the winter-spring cycle (2014). Vertical bars (standard error) (*n* = 3) with different letters are significantly different according to the LSD test (*P* = 0.05).

Although the main color indices (L^*^, h°) were not affected by EC (Table 1), when in FS the EC3.5 compared to EC4.5 increased CHL pigments (Figure 1A).

Considering all these results, it can be argued that in the WS cycle the highest EC level of the NS (EC4.5), in addition to the osmotic stress, could have caused some metabolic disorders, that are likely to be associated with the excessive uptake of Cl^−^ ions. Indeed, the EC4.5 product had the highest level of chloride (+34%) (Table 2), which could have induced negative effects on plant growth, similar to the effect reported for the EFS. In any case, no effect of EC level on tissue physiological disorders, measurable through membrane integrity (EL) and leaf tissue hydration (RWC), was detected (Table 1).

#### Nutritional traits of wild rocket

The increase in EC from 2.5 to 3.5 dS cm^−1^ enhanced the antioxidant profile of the product as a higher concentration of total carotenoids, TPs, AA, DHAA, vitamin C and consequently the HAC and TAC of wild rocket (Table 2). Moreover, EC3.5 also improved the content of the aliphatic EPRO and GLUE and particularly the indolic GBC, while the total GLS concentration did not change (Table 3).

On the contrary, a reduction in concentration of some antioxidant compounds was observed between EC3.5 and EC4.5 (Tables 2, 3): in detail, a reduction in vitamin C, due to a decrease in DHAA (Table 2), and a reduction in total GLS concentration (Table 3), mainly linked to a decrease in aliphatic GRAF, EPRO and GNL. Moreover, no effect of EC level was observed on total carotenoids, TP concentration or TAC (HAC and LAC), which remained unchanged (Table 2).

Several papers confirm the improved qualitative value of leafy species and *Brassicaceae* vegetables associated with a rise in the EC level in the cultivation medium, in terms of higher content of GLSs (broccoli—López-Berenguer et al., 2006, 2008), vitamin C (tomato—De Pascale et al., 2001; *Cichorium spinosum*—Petropoulos et al., 2017), total phenols (sage—Taârit et al., 2012; radish—Yuan et al., 2010; broccoli—Guo et al., 2014), and carotenoids (tomato—De Pascale et al., 2001; lettuce—Kim et al., 2008; Mahmoudi et al., 2010).

The response of plants in terms of the increase in antioxidant compounds with the increase in EC in NS has been explained as a biochemical response to stress conditions to remove the oxidant toxic molecules (De Pascale et al., 2001; Taârit et al., 2012).

However, in agreement with similar experiments on sage (Taârit et al., 2012), cabbage (Sanoubar et al., 2016), and tomato (Krauss et al., 2006), we observed a positive relationship between antioxidant compound levels and medium EC, only up to a threshold (EC3.5) with no further improvements with the highest salinity (EC4.5) (Tables 2, 3), probably due to the concomitant increase in leaf chloride content (Table 2).

Considering the observed effects of NS salinity treatments on AA and DHAA concentration, it seems that the production of AA is promoted by an EC close to 3.5 dS m^−1^, which is likely to be a consequence of the response to the osmotic stress caused by salinity (Guo et al., 2014). On the contrary, higher levels of salinity might have excited more stressful conditions, resulting in an irreversible hydrolization of the DHAA form (Gallie, 2013) and in a loss from the AA pool. As also suggested by Guo et al. (2014), the higher NaCl stress, as underlined by the high Cl concentration in EC4.5-leaves, could have caused an inhibition of the activity of AA regenerating enzyme (Table 2).

With regards to GLS concentration, although no significant improvement in total GLSs was observed passing from EC2.5 to EC3.5, a significant decrease in total GLSs was registered passing from EC3.5 to EC4.5 (Table 3), similar to that observed with the EFS, where temporary and frequent increases in salinity occurred. These results are in agreement with López-Berenguer et al. (2009), when increasing the NaCl levels in NS in broccoli, the GLS content was first increased, with a moderate increase in NaCl in NS, and then decreased at the highest EC values, while leaf chloride content always increased.

By increasing the EC of NS, the GLS qualitative profile showed not unequivocal trends in concentration change for the single GLS (Table 3). As a whole the GLS variation was strictly dependent on two GLSs which were the most abundant (GRAF and GLUE), (Table 3). The total GLS concentration decreased with increasing EC level, mainly due to the decrease in GRAF, while GLUE remained unchanged (Table 3). Conversely in Guo et al. (2013), who examined the effect of several EC levels in broccoli sprouts (up to 100 mol/L NaCl), the decrease in total GLS with increasing EC level, was also due to the decrease in GRAF, while GLUE concentration increased.

It is well-known that GRAF, the most abundant GLSs in rocket (Table 3) and in broccoli (Guo et al., 2013), degrades to isothiocyanate sulphoraphane, an antioxidant and anti-cancer compound.

The retention of TAC under the EC4.5 compared with the EC3.5 regime could be linked to sulphoraphane production, which could cooperate with other antioxidants, such as the phenolic compounds (sulphorafane did not have a direct antioxidant activity) (Guo et al., 2014), so balancing the vitamin C reduction. Further studies on the interactions between NaCl with antioxidants metabolism are needed. As far as nitrate content is concerned, the increase in EC from 2.5 to 3.5 dS m^−1^ resulted in a decrease in nitrate leaf concentration (−47%) (Table 2), along with an increase in DM accumulation (Table 1). These results are in agreement with other experiments on leafy vegetables (Andriolo et al., 2005; Barbieri et al., 2011).

In the WS cycle, the 4.5 dS m^−1^ EC level did not reduce nitrate content compared with the EC3.5 (Table 2) (1,472 mg kg^−1^ FW, on average), although the general climatic conditions of the period were more favorable for nitrogen assimilation (Blom-Zandstra, 1989). It is likely that the higher chloride concentration, occurring in the vacuoles of the EC4.5 grown leaves (Table 2), could have inhibited the activity of the nitrate reductase enzyme (Barber et al., 1989), altering nitrogen metabolism, as above supposed, and resulting in no reduction in nitrate content in leaves.

### Genotype

In both cycles, “Naturelle” performed better than “Nature” in FY (+30%, on average) and DY (+28%, on average) (Table 1), in visual (color) (Table 1) and in nutritional (total GLSs) quality (Table 3). No difference in leaf firmness (DM; SLA) (Table 1), TP or vitamin C content (Table 2) occurred.

The better productive and qualitative performance of “Naturelle” was more evident under less favorable climatic conditions (Tables 1–3).

No interaction between genotype and EC level was detected, while the genotypic response was affected by the SCS, in relation to the cultivation period (Tables 1–3).

**Figure 2:**
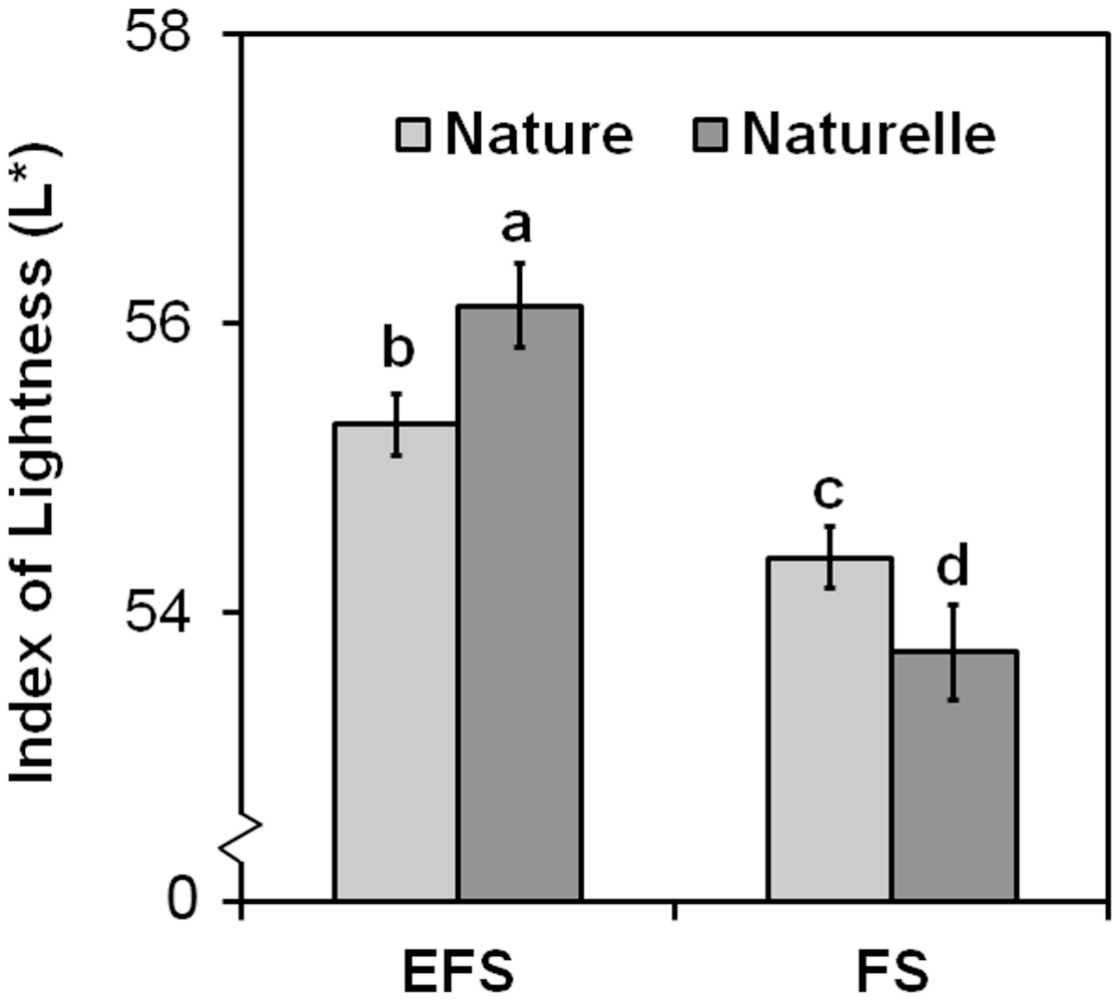
Effect of the soilless cultivation system (FL, Floating system; EFS, Ebb and Flow System) and genotype on brightness index (L^*^) of wild rocket leaves, grown in the winter-spring cycle (2014). Vertical bars (standard error) (*n* = 3) with different letters are significantly different according to the LSD test (*P* = 0.05).

**Figure 3:**
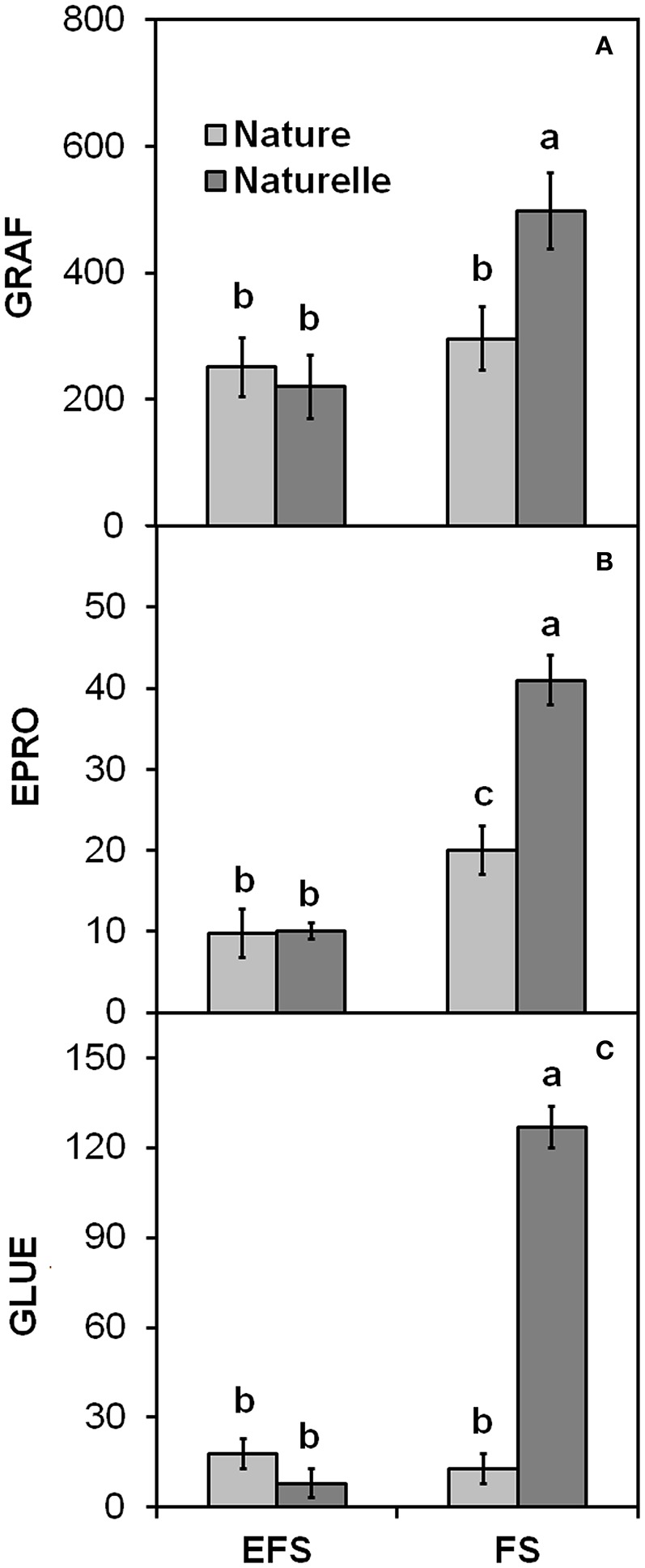
**Effect of soilless cultivation system (FL, Floating system; EFS, Ebb and Flow System) and genotype on (A)** Glucorafanina (GRAF), **(B)** Epiprogoitrina (EPRO) and **(C)** Glucoerucina (GLUE) of leaves of wild rocket, grown in the autumn-winter cycle (2013). Vertical bars (standard error) (*n* = 3) with different letters are significantly different according to the LSD test (*P* = 0.05).

In general, when grown in FS, “Naturelle” performed better than “Nature” in terms of visual quality and nutritional profile. In particular, “Naturelle” showed greener leaves in the WS cycle (Figure 1B—CHL pigments; Figure 2—L^*^ index), while in the AW cycle, it showed the highest value of GRAF, GLUE and EPRO (Figure 3). These latter results support previous studies suggesting that the GLS profile is genotype-dependent (Bennett et al., 2006; D'Antuono et al., 2009; Bell et al., 2015).

“Naturelle” also showed the highest PRO/EPRO ratio, which is strictly correlated with the leaf bitterness and pungency responsible for the unique taste of rocket salad (*Diplotaxis* and *Eruca* spp.) (Pasini et al., 2011).

**Figure 4:**
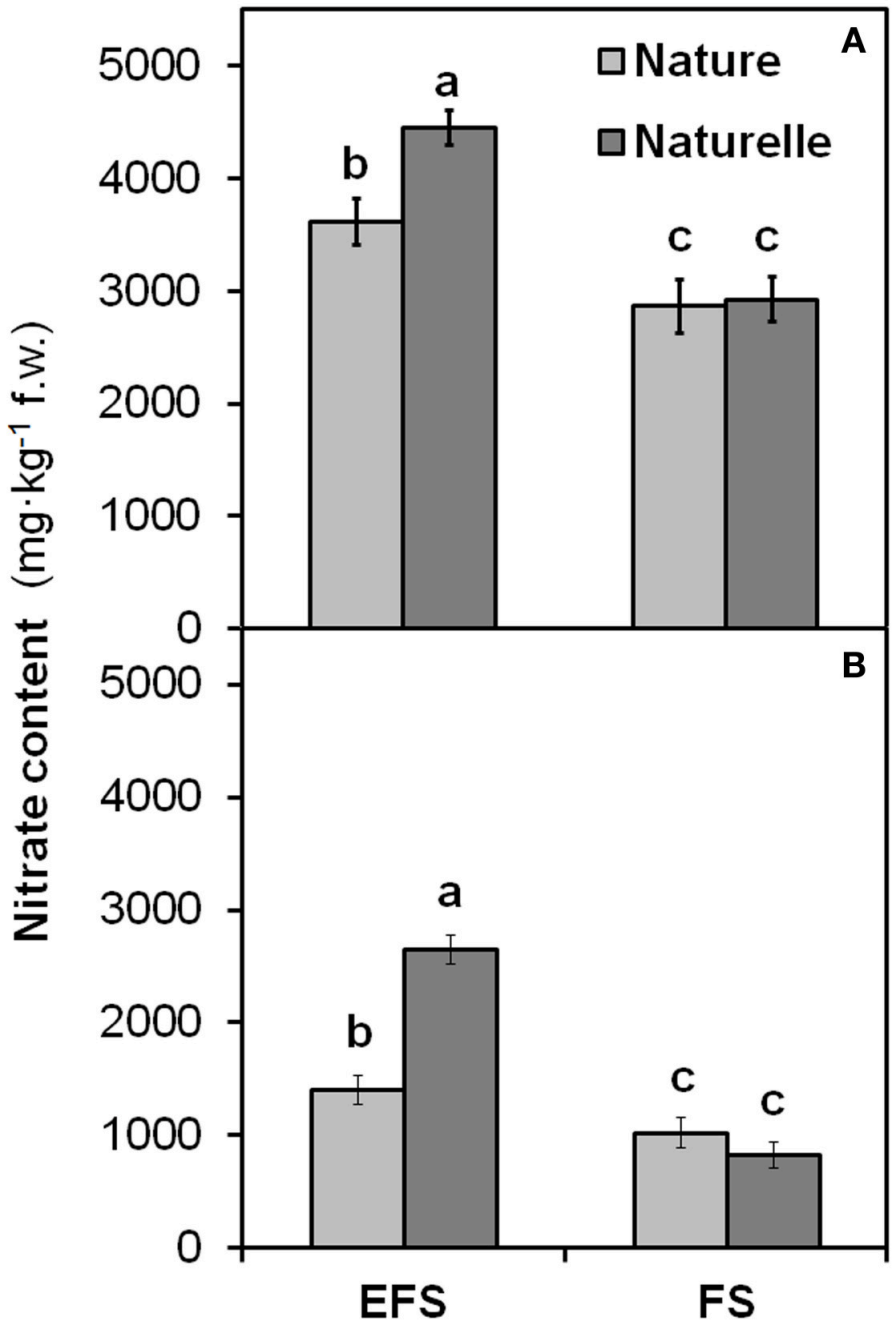
**Effect soilless cultivation system (FL, Floating system; EFS, Ebb and Flow System) and of genotype on nitrate content of wild rocket leaves, grown in the autumn-winter 2013 (A)** and in the winter-spring 2014 **(B)**. Vertical bars (standard error) (*n* = 3) with different letters are significantly different according to the LSD test (*P* = 0.05).

When grown in FS compared with EFS, “Naturelle” showed also a greater reduction in nitrate leaf content than “Nature” (Figure 4), leading us to suppose that “Naturelle,” when not under excessive NaCl stress, uses nitrogen more efficiently than “Nature,” accounting for its better productive performance and improved sensorial, physiological and nutritional characteristics.

## Conclusions

Greenhouse soilless wild rocket salad, when grown in colder periods (autumn-winter in Southern Italy) compared to periods with higher temperature and sunlight (winter-spring in Southern Italy), produces leaves with less fresh and dry mass, less consistence, but with better visual traits. Wild rocket salad obtained during periods with increased temperature and sunlight has an improved nutritional profile linked to its higher content of total phenols, vitamin C, glucosinolates (consequently higher total antioxidant capacity), and lower content of nitrates.

Compared with ebb and flow system (EFS), the floating system (FS) allows better firmness, visual quality and antioxidant profile. FS also produces wild rocket leaves with lower nitrate content than in EFS. Between the two genotypes, “Naturelle” performs better than “Nature,” especially when grown in FS in visual and in nutritional quality.

In both soil cultivation systems, the electrical conductivity (EC) of nutrient solution at 3.5 dS m^−1^ appears to be more appropriate for growing wild rocket. Compared to lower EC, the EC at 3.5 dS m^−1^ enhances leaf consistence, visual quality, and antioxidant compounds, and reduces the nitrate content, without dry weight decrease. Higher EC reduces fresh and dry weight yield, and the antioxidant content. Therefore, compared with low EC levels, a moderate salinity level (3.5 dS m^−1^) improves quantitative and qualitative traits of the crop, while an increase in salinity to higher values (4.5 dS m^−1^) does not improve the physical, physiological or nutritional aspects of wild rocket.

Information from this research may prompt further studies on plant nitrogen and antioxidant compound metabolisms in response to leaf chloride content. In any case, the findings should be useful to growers when making soilless management decisions for improving the yield and the quality of wild rocket, such as its suitability for fresh-cut processing and its phyto-chemical profile.

## Author contributions

AB: Conception of the work, Data collection, Data analysis, and interpretation, Drafting the article; CL: Design of the work, Data collection, data analysis; GC: Conception of the work, Data analysis and interpretation, Drafting the article, Critical revision of the article, Final approval of the version to be published; AE: Conception of the work, Critical revision of the article, Final approval of the version to be published.

## Funding

The research leading to these results has received funding from the European Union's Seventh Framework Programme for research, technological development and demonstration under grant agreement n 289719 (Project QUAFETY).

### Conflict of interest statement

The authors declare that the research was conducted in the absence of any commercial or financial relationships that could be construed as a potential conflict of interest.
