# Supplementary figures and images for: Nutritional, Biophysical and Physiological Characteristics of Wild Rocket Genotypes As Affected by Soilless Cultivation System, Salinity Level of Nutrient Solution and Growing Period
Source: Front Plant Sci. 2017 Mar 9;8:300. doi: 10.3389/fpls.2017.00300 (PMC5343037; doi:10.3389/fpls.2017.00300)

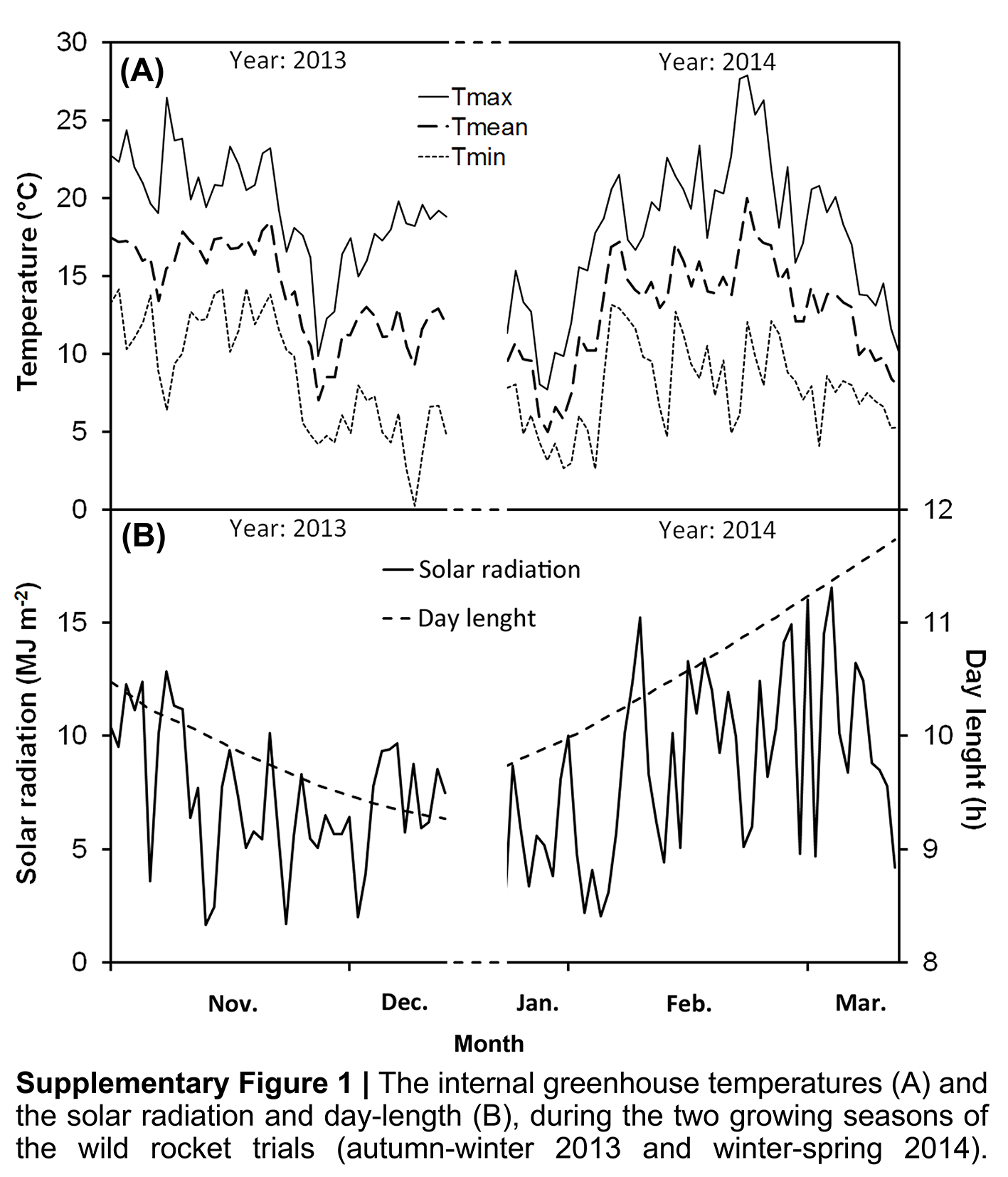

Supplement: Supplementary file 1 [file Image1.TIF]
